# Antibiotic treatment for intermittent bladder catheterisation with once daily prophylaxis (the AnTIC study): Study protocol for a randomised controlled trial

**DOI:** 10.1186/s13063-016-1389-y

**Published:** 2016-06-04

**Authors:** Catherine Brennand, Alexander von Wilamowitz-Moellendorff, Sarah Dunn, Jennifer Wilkinson, Thomas Chadwick, Laura Ternent, Yemi Oluboyede, Ruth Wood, Katherine Walton, Mandy Fader, James N’Dow, Mohamed Abdel-Fattah, Doreen McClurg, Paul Little, Paul Hilton, Anthony Timoney, Nicola Morris, Nikesh Thiruchelvam, James Larcombe, Simon Harrison, Heather Armstrong, Elaine McColl, Robert Pickard

**Affiliations:** Newcastle Clinical Trials Unit, Newcastle University, 1-2 Claremont Terrace, Newcastle upon Tyne, NE2 4AE UK; Institute of Health and Society, Baddiley Clark Building, Newcastle University, Richardson Road, Newcastle upon Tyne, NE2 4AX UK; Department of Medical Microbiology, Freeman Hospital, The Newcastle upon Tyne Hospitals NHS Foundation Trust, Newcastle upon Tyne, UK; University of Southampton, Southampton General Hospital, Tremona Road, Southampton, SO16 6YDUK UK; Academic Urology Unit, University of Aberdeen, Health Sciences Building (2nd floor), Foresterhill, Aberdeen, AB25 2ZD UK; Glasgow Caledonian University, Cowcaddens Road, Glasgow, G4 0BA UK; Aldermoor Health Centre, Aldermoor Close, Southampton, SO16 5ST UK; The Newcastle upon Tyne Hospitals NHS Foundation Trust, Newcastle upon Tyne, UK; Department of Urology, Southmead Hospital, North Bristol NHS Trust, Bristol, SB10 5NB UK; Addenbrooke’s Hospital, Cambridge University Hospitals NHS Trust, Hills Road, Cambridge, CB2 0QQ UK; Harbinson House Surgery, Front Street, Sedgefield, Co Durham TS21 3BN UK; Mid-Yorkshire NHS Trust, Pinderfields Hospital, Aberford Road, Wakefield, West Yorkshire WF1 4DG UK; Patient advisor, Durham, United Kingdom; The Medical School, Newcastle University, Newcastle upon Tyne, UK; Institute of Cellular Medicine, The Medical School, Newcastle University, 3rd Floor, William Leech Building, Framlington Place, Newcastle upon Tyne, NE2 4HH UK

**Keywords:** Self-catheterisation, Antibiotic prophylaxis, Urinary tract infection, Randomised controlled trial, antibiotic resistance, UTI, RCT

## Abstract

**Background:**

Clean intermittent self-catheterisation is an important management option for people who cannot empty their bladder effectively. Recurrent urinary tract infections are common in these patients. Data from recent studies suggest that antibiotic prophylaxis may be beneficial in reducing infection risk, but the effectiveness of this intervention remains uncertain.

**Methods/design:**

This is a 52-site, patient randomised superiority trial set in routine care comparing an experimental strategy of once daily antibiotic prophylaxis for 12 months against a control strategy of no prophylaxis in people who carry out self-catheterisation and suffer recurrent urinary tract infections. The primary outcome is number of urinary tract infections during a 12-month treatment period. Both groups will otherwise receive usual care including on demand treatment courses of antibiotics for urinary tract infection. Participants and their clinicians will not be blinded to the allocated intervention, but central trial staff managing and analysing trial data will, as far as possible, be unaware of participant allocation. The analysis will follow intention-to-treat principles.

**Discussion:**

This trial was commissioned and funded by the United Kingdom National Health Service following prioritisation of the research question by the National Institute for Health and Care Excellence.

**Trial registration:**

ISRCTN67145101 EUDRACT2013-002556-32. Registered on 25 October 2013.

**Electronic supplementary material:**

The online version of this article (doi:10.1186/s13063-016-1389-y) contains supplementary material, which is available to authorized users.

## Background

Clean intermittent self-catheterisation (CISC) is a frequently used intervention for people who cannot empty their bladder effectively due to bladder outlet obstruction or failure of bladder muscle contraction which may be associated with neurological disease [1]. Patients needing CISC are taught how to insert a catheter, drain the bladder and then remove the catheter [2]. Single-use disposable catheters, typically with a hydrophilic coating, are predominantly used in the UK [3].

There are no accurate prevalence data for CISC use in the UK. The National Health Service (NHS) England prescription database recorded 47 million CISC catheter prescriptions in 2010 at a cost of £64 million [4]. If we assume each individual uses an average of 25 catheters per week, this suggests that there are about 36,000 CISC users in England and perhaps 43,000 in the UK as a whole. This estimate is in agreement with a primary care-based estimate which found a prevalence of approximately 7–8 per 10,000 adults, suggesting an approximate total of 43,000–49,000 adults in the UK (Fader M., personal communication, August 2011).

Recurrent urinary tract infection (UTI) is the commonest adverse event experienced by CISC users affecting between 12–88 % of studied cohorts [5]. Differentiation between asymptomatic bacteriuria, which would not normally be treated, and symptomatic UTI in these studies is difficult. One estimate suggests that 50 % of users have persistent bacteriuria and at least 25 % suffer two or more symptomatic UTI episodes per year [6]. Conservatively this suggests that up to 10,000 CISC users in the UK suffer recurrent UTI, the target population for this trial. Neurological disease, female sex, young age and high bladder volumes are associated with higher prevalence of UTI [1]. *Escherichia coli* (*E. coli*) is the causative bacterium in 60–70 % of cases [6]. Most episodes are associated with transient symptoms such as lower abdominal pain, urethral pain and flu-like symptoms; occasionally systemic upset can occur with fever and loin pain. Those with reduced bladder sensation may alternatively complain of cloudy urine, increased odour and incontinence [7]. Recurrent UTI is distressing and an additional burden for patients with underlying neurological disease and functional disability [5]. For some there is a risk of renal damage in the longer term [7] or significant sepsis requiring intensive care and potential mortality.

A number of simple interventions have been trialled to reduce UTI risk for CISC users, including single-use and hydrophilic catheters and antiseptics, but none showed efficacy [2]. A randomised controlled trial (RCT) reported a benefit of hydrophilic catheters for patients with spinal cord injury during initial hospitalisation [8]. The need for strategies to reduce the prevalence of UTI in this population has been emphasised by reports in the UK from the James Lind Alliance and the National Institute for Health and Care Excellence (NICE) [9, 10].

Once daily low-dose antibiotic prophylaxis is effective for women without bladder emptying problems who suffer simple recurrent UTI. Systematic review and meta-analysis of trials in this patient group showed a relative risk for UTI (95 % confidence interval, CI) against placebo of 0.15 (0.08–0.28) [11]. Adverse events in trials using nitrofurantoin, trimethoprim or cephalexin were mild and rarely associated with withdrawal, but were more frequent in the antibiotic group with a relative risk (95 % CI) of 1.78 (1.06–3.0); gastro-intestinal upset, skin rash and vaginal candidiasis predominated. Nitrofurantoin appeared more effective than trimethoprim but resulted in more withdrawals. These two drugs together with cephalexin are recommended and licensed for this purpose in the UK [12]. There were no reports of serious adverse effects such as neuropathy or pulmonary fibrosis in the nitrofurantoin arms of randomised studies included in the Cochrane review, but an observation study of prophylactic nitrofurantoin noted one episode of possible neuropathy in 219 patients over 12 months’ use [13].

Current evidence for effectiveness of antibiotic prophylaxis by CISC users, the focus for this trial, has been summarised by a Cochrane review updated to September 2011 [14]. We were unable to identify any further published or ongoing trials from that date up to December 2015. The review found six RCTs involving adults (four trials) or children (three trials) performing CISC for neurological bladder dysfunction with a total of 406 participants. Five had placebo as a comparator with either a crossover or parallel group design, and three each used clinical or microbiological definition for UTI outcome; the latest report was 2011. Two of the crossover trials had a duration of 3 months for each intervention without washout whilst one had a duration of 5 months for each intervention and a 1-month washout period. The prophylactic agents used were trimethoprim-sulfamethoxazole (two trials) and nitrofurantoin (four trials) and a variety of agents (one trial). Participant attrition after randomisation ranged from 2–35 %. One trial using the outcome of clinically defined symptomatic UTI found a relative incidence rate (95 % CI) of 0.50 (0.17–1.44) in favour of antibiotic prophylaxis whilst another trial found no difference. For the outcome of microbiologically proven symptomatic UTI, one trial found a relative risk of 0.78 (0.62–0.79) in favour of prophylaxis. Evidence from four trials showed an overall relative incidence rate (95 % CI) for bacteriuria of 0.61 (0.44–0.87) in favour of prophylaxis. The review authors concluded that, although results were promising, there was a lack of unequivocal evidence for effectiveness of antibiotic prophylaxis for CISC users, agreeing with a previous review [15]. Recommendations for future trials were as follows:

- Use incidence of symptomatic UTI as the primary outcome
- Measure antibiotic resistance
- Control for factors increasing UTI risk: sex, frequency of catheterisation, neurological cause, frequency of previous UTI, prior use of antibiotic prophylaxis

None of these trials found any excess harms in the prophylaxis groups, but ecological changes in pathogens were not studied. These results and the need for further trials have been highlighted in a further narrative review [16].

In a large RCT of antibiotic prophylaxis of recurrent UTI in women with normal voiding, it was found that faecal and urinary carriage of resistant *E. coli* was increased from 40 % to 80 % by use of trimethoprim-sulfamethoxazole once daily prophylaxis but that this returned to baseline 3 months after discontinuing of the antibiotic prophylactic therapy [17]. There remains public health concern regarding the empiric prophylactic widespread use of antibiotics given the rapid emergence of resistant strains of bacteria, including *E. coli*. Antimicrobial stewardship, including the appropriate use of antimicrobial prophylaxis, has become a priority.

This background has led us to design a robust pragmatic trial to determine whether the apparent benefit of antibiotic prophylaxis seen in small trials amongst specific groups of CISC users is also seen in a routine care setting and whether the benefits are worthwhile in terms of harms. The estimates of prevalence, effectiveness and harms have allowed us to power the trial conservatively based on what we consider to be a minimum important difference from clinician, patient and economic perspectives.

## Methods/design

This is a 52-site, pragmatic, patient randomised superiority trial designed to answer the question: In people carrying out CISC who suffer recurrent UTI does an experimental strategy of once daily antibiotic prophylaxis reduce the rate of symptomatic UTI compared to a control strategy of no prophylaxis? Both groups will otherwise receive usual care, including on demand discrete courses of antibiotic treatment for UTI. The trial will be set in both primary and secondary National Health Service (NHS) care in the UK. Participants and their clinicians will not be blinded to the allocated intervention, but central trial staff managing and analysing trial data will, as far as possible, be unaware of participant allocation. We will also assess participant perception of benefit: firstly, by completion of a treatment satisfaction questionnaire on exit; secondly, by qualitative analysis of semi-structured interviews on trial completion exploring the views and attitudes of a purposive sample of participants towards the trial intervention. The primary economic analysis will assess the cost per UTI avoided, but we will also perform a cost-utility analysis and a contingent valuation study. Bacterial ecological change will be assessed by comparing changes in resistance patterns of *E. coli* in participant urine and perianal swab samples. We have formulated a recruitment plan to progressively build to a target of 372 participants over 28 months. Outcomes will be collected over 12 months for each participant and analysed at trial termination according to intention-to-treat principles.

The null hypothesis is that the effectiveness and cost-effectiveness of a strategy of prophylactic antibiotic are not superior to those for no prophylaxis over 12 months.

**Fig. 1:**
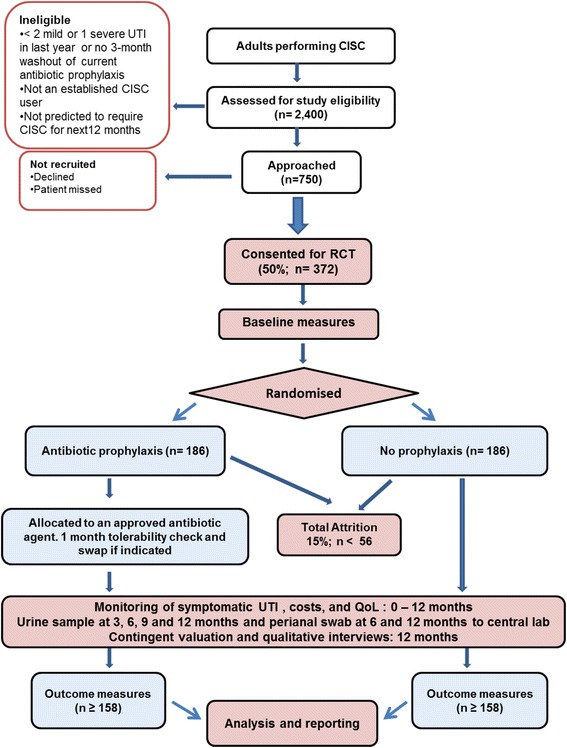
CONSORT diagram showing flow of trial participants through the trial

The anticipated trial flow for participants is illustrated in Fig. 1.

### Objectives

#### Primary objectives

The primary objectives are to:

- Determine the relative impact of each intervention on incidence of UTI over 12 months
- Determine the incremental cost per symptomatic UTI avoided

#### Secondary objectives

The secondary objectives are as follows:

- Clinical
  - ○ Determine the relative effect on quality of life (QoL) amongst trial participants
  - ○ Measure overall satisfaction with prophylactic antibiotic treatment
  - ○ Assess participants’ perception of benefit at 12 months using qualitative methodology
  - ○ Record adverse effects related to both prophylaxis and treatment antibiotic use
  - ○ Determine relative rates of hospitalisation because of UTI
  - ○ Measure difference in renal function by estimated glomerular filtration rate (eGFR) at 12 months
  - ○ Determine rates of asymptomatic bacteriuria at 12 months
  - ○ Assess ecological change in *E. coli* isolated from urine and perianal swabs
- Economic
  - ○ Measure incremental cost per QALY gained through repeated completion of SF-36
  - ○ Assess participants’ willingness to pay to avoid a UTI by contingent valuation at end of trial participation and incorporate these data in the economic evaluation using a cost-benefit framework.

### Primary outcome measures

The primary outcome is difference in incidence of symptomatic UTI during the 12-month observation period. Symptomatic UTI will be defined on fulfilment of two criteria. The first will be the presence of at least one patient-reported or clinician-recorded symptom from a predefined list encompassing the recommendations of the British Infection Association (BIA) [18], the Centers for Disease Control and Prevention (CDC) [19] and spinal cord injury UTI consensus statement [8] comprising: fever (being hot and sweaty); shivers; cloudy urine; smelly urine; visible blood in urine; new or increased urinary leakage (incontinence); lower abdominal pain; having to catheterise more often; having to rush to catheterise; pain when one puts the catheter in; feeling generally unwell (‘fluey’); stiffness or worsening stiffness (spasticity) of arms and legs. The second criterion is taking a discrete treatment course of antibiotics prescribed by a clinician or as part of a patient-initiated self-start policy. Occurrence of symptomatic UTI with prescription of a treatment course of antibiotic will be captured by:

- Participant log with report alert sent by participant to trial staff.
- Contact with each participant at least every 3 months by local trial staff and more frequently if required to aid participant recording of UTI episodes.
- Response to specific enquiry in participant questionnaire completed at 3, 6, 9 and 12 months.
- End of trial review of hospital and primary care record at 12 months.
- For any identified treatment course of antibiotics for UTI the participant will be asked to complete a multiple choice description of symptoms that precipitated the request for antibiotic treatment.

To ensure consistent attribution we will set a hierarchy of evidence on which to base the primary outcome. First will be participant-reported episodes of symptoms that they considered to be due to UTI and for which they obtained treatment with an appropriate antibiotic. If, in discussion with the participant, there is uncertainty as to whether an antibiotic was taken or if the stated antibiotic was not of a type normally used for UTI, the relevant general practitioner (GP) or hospital record will be checked for confirmation that a prescription for an antibiotic to treat UTI was issued (including previous prescriptions for self-start therapy). Where no antibiotic prescription was found in the record, we will ask the participant to confirm the origin of the prescription. If we were unable to confirm issuing of either a single course or self-start supply of antibiotics, then the primary outcome will not be fulfilled. The second type of event will be the identification of a prescription of an antibiotic during the planned 3-monthly interrogation of healthcare records without a participant report of a UTI. In this case the participant will be contacted to check that they did take a treatment course of antibiotics at that time and to assess their symptom status. If the participant had no recollection of the antibiotic course, or if there was no evidence from the participant or healthcare records of any change to baseline urinary symptoms, then the episode will be judged not to have fulfilled the primary outcome.

#### Primary economic outcome

The primary economic outcome is the incremental cost per symptomatic UTI avoided. It includes the following costs associated with the prophylaxis and no prophylaxis strategies including cost of harms:

- ○ Treatment costs of drugs and healthcare services from standard NHS sources such as the British National Formulary (BNF) and published tariffs from NHS reference costs
- ○ Health resource utilisation questionnaire at baseline and at 6 and 12 months
- ○ Patient costs from a time and travel questionnaire as part of the 12 months exit assessment

### Secondary outcome measures

Secondary outcome measures will be collected as additional criteria to the primary outcome from inspection of participant healthcare records, by participant questionnaire or by clinical test performed at specific time points. The following are also included.

- The rate of UTI will also be alternatively defined as the *incident density rate*; the number of UTIs suffered during the observation period minus days spent taking treatment courses of antibiotics active against urinary tract organisms.
- *Febrile UTI* defined as the primary outcome plus presence of a recorded fever of more than 38 °C:
  - ○ Confirmed by inspection of primary or secondary healthcare record by research staff.
- *Microbiologically confirmed symptomatic UTI* defined as the primary outcome plus positive urine culture:^1^
  - ○ Participants will provide an intermittent catheter specimen of urine for local analysis as requested by the treating clinician. This will be analysed according to clinician decision by the local microbiology using the local standard operating procedure (SOP). We will also ask the participant to send a urine specimen using provided safe packaging to the central microbiology trial laboratory in the Newcastle upon Tyne Hospitals NHS Foundation Trust each time they consider they have symptoms suggestive of UTI and intend to commence a course of antibiotic treatment. This will be analysed and cultured on receipt and the result used for trial outcome purposes but not for patient care.
- *Antibiotic prescription for asymptomatic UTI* without participant-reported or clinician recorded evidence of symptom change.
- *Asymptomatic bacteriuria* defined as a positive urine culture in the absence of symptoms.
  - ○ Participants will be asked to send a urine sample to the central laboratory at baseline prior to randomisation and during asymptomatic periods in months 3, 6, 9 and 12 of their trial participation. They will also be separately consented to provide a urine sample 6 months after completion of trial (18-month time point).
- *Hospitalisation due to UTI* defined as an unplanned visit to hospital for treatment of a UTI which required at least one overnight stay in hospital.
  - ○ Collected from healthcare record review and checked from participant report or enquiry.
- *Participant perception of benefit.*
  - ○ We will record and analyse semi-structured interviews with up to 30 participants purposively sampled from both trial arms on completion of their 12-month trial period.
- *Overall satisfaction with allocated treatment strategy.*
  - ○ Participants will complete the treatment satisfaction questionnaire for medication at 12 months as part of their completion of trial questionnaire [20].
- *Generic health-related quality of life.*
  - ○ Participant completion of the SF-36 1-week recall questionnaire at baseline, 3, 6, 9 and 12 months and within the first 2 days of each episode of symptomatic antibiotic-treated UTI [21].

#### Adverse effects (harms)

The adverse effects are as follows.

- *Adverse effects of antibiotic therapy*
  - ○ During antibiotic prophylaxis use
    - ▪ Collected by participation checklist completed at 3, 6, 9 and 12 months
    - ▪ Recording of relevant data from primary and secondary care records at 12 months
  - ○ During treatment antibiotic use
    - ▪ Collected as part of participant log during episode of antibiotic-treated UTI
    - ▪ Recording of relevant data from primary and secondary care records at 12 months
- *Change in renal function* defined as estimated creatinine clearance measured by serum creatinine blood test at baseline before randomisation and during an asymptomatic period in month 12
- *Change in liver function* defined as clinically significant change in liver function indices measured by serum liver function tests (LFTs) at baseline and at 12 months
- *Bacterial ecological changes* to type and resistance patterns of *E. coli* isolated from urine specimens and perianal swabs analysed at the central laboratory at baseline prior to randomisation and at 3, 6, 9 and 12 months after randomisation and from surveillance of other urine specimens submitted to the central laboratory. Participants will also be separately consented to provide a urine specimen and perianal swab together with an antibiotic use questionnaire 6 months after completion of trial (18-month time point).

#### Secondary economic outcomes

The secondary economic outcomes are as follows:

- Incremental cost per QALY gained
  - ○ QALY estimated from responses to repeated administration of the SF-36 as described above
- Participants’ willingness to pay (WTP) to avoid a UTI
  - ○ Measured by completion of a contingent valuation questionnaire at end of month 12

### Study design and duration

#### Intervention

This trial is pragmatic in design and, apart from randomisation to prophylaxis/no prophylaxis strategies and collection of outcome data, participant care will follow standard pathways in participating UK NHS sites across both primary and secondary care. Participants in both trial groups will receive discrete courses of antibiotics as decided by the responsible clinician for symptomatic UTI.

#### Antibiotic prophylaxis (experimental)

The experimental intervention is the use for 12 months of a once daily low oral dose of an antibiotic active against common urinary pathogens. The agent to be used will be selected by the responsible clinician depending on patient characteristics such as previous use, allergy, possibility of future pregnancy, renal function, prior urine cultures and local guidance. There is no universally agreed upon national guidance, but available evidence suggests use of nitrofurantoin 50 mg (or 100 mg dependent on participant weight), trimethoprim 100 mg or cephalexin 250 mg, in that order of preference [23–25]. Renal function will be determined by estimated glomerular filtration rate (eGFR) at baseline, and if this is less than 45 mL/min nitrofurantoin will not be used. Otherwise participants and their clinicians will be asked to review the prescribing information for each drug given in the trial documentation to guide selection of the most appropriate initial agent. At the planned 1-month telephone review, local trial staff will ask about tolerability of the prescribed medication. If there are specific and intolerable adverse effects, then switching to an alternative agent would be advised in consultation with the relevant clinician with the reasons for the change recorded. This process would then be repeated at planned 3-monthly reviews and a third agent advised if necessary.

More frequent telephone follow-up will be undertaken if needed to help the participants become established on a suitable agent. The aim will be to maintain participants allocated to the prophylaxis group on prophylaxis for as long as possible during the 12-month trial period within tolerance and safety constraints. Participants will be asked to take the once daily antibiotic prophylaxis as a single dose at bedtime. If participants in the prophylaxis group develop symptoms and signs suggestive of breakthrough UTI, then they will seek treatment in their usual way, predominantly by contacting their GP and starting a discrete treatment course of antibiotics. In this scenario they will be instructed to stop the prophylactic antibiotic whilst they are taking a treatment course and restart it again the day following the last dose they take of the treatment course. Clinicians and participants will be advised to use a different agent for treatment than the one they are taking for prophylaxis. Details of all treatment antibiotic courses, including the agent used and the number of days participants actually took the prescribed antibiotic, will be recorded in the patient log and trial case report form (CRF). All adverse events will also be recorded.

#### No prophylaxis

The control arm will be a strategy of no prophylaxis. Participants will self-monitor their symptoms as usual and report to their GP if they develop symptoms and signs suggestive of UTI requiring treatment.

#### Standard care for both groups

Apart from the randomised allocation to prophylaxis and the avoidance of the prophylactic agent as treatment for symptomatic UTI, there will not be any differences in the trial protocol concerning care of experimental and control groups of participants. We will ensure as far as is possible that participants in both groups receive their usual care in terms of identification and treatment of UTI, health surveillance and support related to use of CISC, and monitoring and treatment of the underlying cause of their lower urinary tract dysfunction. For purposes of generalisability of trial results and to input into relevant trial outcomes and sub-group analysis, we will record all healthcare episodes for each participant. We consider standard care for users of clean intermittent self-catheterisation (CISC) who suffer recurrent UTI to be the use of discrete treatment courses of antibiotics as indicated by symptoms or signs of UTI.

Treatment will typically involve a 3- or 7-day course of an antibiotic active against urinary pathogens depending on severity of symptoms. A urine specimen would normally be sent for microbiological examination at the time of starting antibiotic treatment. If therapy was successful no further action would be required, whereas if symptoms did not resolve the choice and duration of antibiotic would be reconsidered in the light of urine culture result and if necessary a further urine sample submitted for analysis [18]. Regular renal surveillance using serum creatinine and ultrasound would also be expected. Guidance will be provided to participants in both groups, and their clinicians regarding the use of urine testing and antibiotic options in terms of agents used and their duration. Participants in both groups will continue their regular care with primary and secondary care clinic visits, access to continence advice and relevant patient support groups according to local practice and individual preference.

#### Delivery of interventions

Local clinicians at the site of randomisation will be responsible for initiating trial medication for those participants allocated to prophylaxis. If the participant wishes and if the clinician responsible for their routine care agrees, then the antibiotic prophylaxis can be continued beyond the 12-month trial participation period but without further active monitoring for trial purposes.

#### Definition of end of study

The end of study is the last participant’s final study contact at 12 months after his randomisation. We will separately consent at baseline for the sending of urine and perianal swab specimens at 6 months after end of study (18-month time point) together with completion of an antibiotic usage questionnaire. This is done to determine the final bacterial ecology outcome.

#### Sources of bias

To allow randomisation, eligible participants and the responsible clinician will both need to be sufficiently uncertain of whether the experimental or control strategy is better for relief of recurrent UTI considering each individual’s particular circumstances. Given the lack of high-level evidence as to which is more effective, we will provide trial information illustrating the uncertainty and the need for a definitive trial. This will act to ensure that any selection bias in terms of characteristics of CISC users willing to be randomised is minimised. An anonymised screening log will be kept at each site listing demographic and clinical characteristics and reasons for declining randomisation (if offered) and used for comparison of this group with those entering and those completing the trial. Secondly the characteristics of participants who cross over at randomisation or at a later stage, withdraw or regret their allocated treatment option may differ from those completing 12 months of their allocated strategy. We will address this by comparison of SF-36 scores between these groups measured at baseline prior to randomisation and following treatment. Trial literature given to all participants and to their clinicians will detail other measures to reduce the risk of UTI such as adequate fluid intake, increased frequency of catheterisation, cranberry products and, if appropriate for post-menopausal women, vaginal oestrogen supplements.

Given the lack of blinding, it is possible that participants allocated to the control of no prophylaxis will be more likely to seek treatment for symptoms suggestive of UTI, and that their clinicians may be more likely to prescribe treatment antibiotic, thus introducing bias to our primary outcome. To reduce the potential for this bias, we will give information on the use of antibiotic treatment, describing indication and choice of agent, in trial literature to participants and their GPs according to established guidance from the British Infection Association (BIA) and other groups. We will also include in the participant information packs advice on when to seek help regarding symptoms suggestive of UTI and the use of simple measures to avoid or avert symptomatic UTI. To ensure uniformity of processing and culture techniques, we will use the results of analysis performed at our central laboratory for trial outcomes. The local result will be used for the study if the central result is missing.

#### Target population and sample size

We will recruit from the population of adult users of CISC. The setting is NHS hospitals and community sites throughout the UK where CISC use is taught and monitored. We expect to randomise at least 372 participants over a 28-month period. For primary outcome purposes, follow-up will continue for 12 months after randomisation. Participants will be given the option of submitting an additional urine and perianal swab sample 6 months after trial completion (18-month time point) to assess return to baseline of *E. coli* ecology. Separate consent will also be asked for permission to access clinical records for extended follow-up for a further 9 years (10 years in total) and for life-long linkage to central NHS databases.

#### Target population

The target population consists of adult established CISC users predicted to continue its use for at least 12 months who have:

1. Suffered at least two episodes of CISC-related symptomatic oral antibiotic-treated UTI within the previous 12 months managed in the community or
2. Suffered at least one episode of severe CISC-related symptomatic parenteral antibiotic-treated UTI within the previous 12 months requiring hospital care or
3. Previously received prophylactic antibiotic therapy for recurrent symptomatic UTI within the 12 months prior to starting prophylactic antibiotic therapy and who have completed a 3-month washout period without taking antibiotic prophylaxis prior to randomisation.

### Inclusion criteria

The inclusion criteria are as follows:

- Adult men and women aged ≥18 years
- Completed training of CISC and predicted to continue use for at least 12 months
- Able to give informed consent for participation in trial
- Able and willing to adhere to a 12-month follow-up period
- Have either suffered at least two episodes of symptomatic UTI related to CISC within the last 12 months, or at least one episode of UTI requiring hospitalisation, or for those previously prescribed prophylactic antibiotic for UTI, have completed a 3-month washout period without antibiotic prophylaxis. Any active symptomatic UTI will be treated prior to randomisation
- Able to take a once daily oral dose of at least one of nitrofurantoin, trimethoprim or cephalexin
- Intermittent catheterisation may be performed by participant, spouse or carer
- No restriction on type of catheter used

### Exclusion criteria

The exclusion criteria are as follows:

- Age <18 years
- In learning phase of CISC
- Already taking prophylactic antibiotic against UTI and declining 3-month washout period without antibiotic prophylaxis (this will be specifically monitored in the screening log)
- Inability to take any of the three prophylactic antibiotic agents due to multiple drug sensitivities
- Women who intend to become pregnant during planned period of trial participation or who are pregnant or who are breastfeeding
- Previous participation in this study
- Inability to give informed consent or have primary outcome information collected

### Screening recruitment consent

#### Participant identification and invitation to participate

We will ask clinical staff at each site to identify eligible participants through direct contact or by searches of electronic records. Identified potentially eligible patients will be sent brief details of the need and purpose of the study and eligibility criteria. This will emphasise the pragmatic nature of the study and give a realistic indication of the burden to participants. Contact details of central and local trial teams will be provided so that interested patients can express a willingness to know more about the project and agree to be contacted by the research team. Once identified and agreeing to be contacted by the research team, eligible potential participants will be contacted by trial staff and if willing will be sent or given the trial participant information material. This recruitment activity will be coordinated centrally but administered at each site.

All subjects who agree to participate will be seen by local research staff in order to go through the consent and randomisation procedure. A screening log will be kept at each site to document details of subjects invited to participate in the study. Non-identifying patient details to allow assessment of selection bias such as age, number of episodes of UTI in past 12 months, previous use of antibiotic prophylaxis for UTI, type of bladder dysfunction and type of catheter used will be uploaded to the secure study website for subsequent analysis. For subjects who decline participation, the log will document reason for non-participation. The log will also ensure potential participants who are ineligible or decline participation are approached only once. Participants who do not respond after being sent or given written information about the study may be contacted a second time to ensure they have received the information and been given the opportunity to participate.

#### Consent procedures

An informed consent discussion will be undertaken by site staff authorised in the delegation log. This will include medical staff and research nurses involved in the study who will give time for participants to ask any questions they may have following review of the trial information pack. Following receipt of information about the study, participants will be given at least 24 hours and up to as much time as they need to decide whether or not they would like to participate. Those wishing to take part will provide written informed consent by signing and dating the study consent form, which will be witnessed and dated by a member of the research team with documented, delegated responsibility to do so. Written informed consent will always be obtained prior to randomisation. The original signed consent form will be retained in the Investigator Site File, with a copy filed in the clinical notes, a copy given to the participant and a copy faxed to the central trial office. Participants will specifically consent to their GP being informed of their participation in the study. The right to refuse to participate without giving reasons will be respected.

#### Subject withdrawal

Patients will remain in the study unless they withdraw consent or in the unlikely event that the local Principal Investigator (PI), Chief Investigator (CI) or trial office feel it is no longer appropriate for the patient to continue. If a participant chooses to withdraw, we will seek continuation of consent for collection of outcome data from clinical records.

### Randomisation

#### Participant allocation

Randomisation will be administered centrally by the Newcastle Clinical Trials Unit secure web-based system. Permuted random blocks of variable length will be used to allocate participants 1:1 to the control and experimental groups and ensure concealment of allocation from central trial staff. An individual not otherwise involved with the study will produce the final randomisation schedule. Stratification by three variables: (1) prior frequency of UTI: <4 episodes per year and ≥4 episodes per year, (2) a diagnosis of neurogenic lower urinary tract dysfunction: yes or no and (3) sex: female or male, will be performed prior to randomisation to ensure balanced allocation within these factors. For those allocated to prophylaxis an appointment will be arranged, facilitated by trial staff, with the prescribing clinician to commence prophylaxis. This may be a hospital consultant, a GP or a nurse specialist. Continued supply will be ensured typically through repeat prescription from the individual participant’s GP.

Patients may only be randomised into the study by an authorised member of staff at the study research site, as detailed in the delegation log.

### Blinding

Assignment to either prophylaxis or no prophylaxis will not be blinded to either the participant or investigator or the local research staff (a non-blinded study). However, central trial staff responsible for data management, entry and analysis will be unaware of allocated intervention as far as possible.

### Data collection and follow-up

Outcome data will be recorded by the participant on questionnaires, which will be made available in paper-based and electronic format, and on the worksheet by local research staff under a unique identifier with subsequent electronic entry onto electronic case report forms (eCRFs) onto the web-based secure clinical data management system for storage at Newcastle Clinical Trials Unit (CTU). Baseline data will include demographics, underlying disease characteristics, details of catheterisation, prior frequency of UTI and associated usage of healthcare and past urine microbiological reports, together with symptom and QoL measures recorded prior to randomisation. Urine, blood and perianal swab samples will also be collected at baseline for immediate testing and banking of serum, urine and *E. coli* isolates for additional studies. During the 12 months of study participation, participants will be asked to keep a simple log recording episodes of suspected UTI from a symptom and help-seeking point of view. If required, trial staff will contact participants approximately monthly to help complete the UTI logs within a reasonable recall window. The patient logs will be validated if necessary by data from regular inspection of primary and secondary healthcare records for UTI events and subsequent direct checking with participants by their preferred means (telephone, text, e-mail). Other outcome data will be collected by patient questionnaire at 3, 6, 9 and 12 months supplemented by regular inspection of health records. Details of participant progress will be recorded on CRFs. A schedule of events is shown in Table 1.

**Table 1:** Schedule of study interventions and outcome data collection from participant

Intervention/data	Visit 1 Initial screen	Visit 2	Visit 3	Visit 4	Visit 5	Visit 6	At time of UTI
		Consent, baseline and randomisation	3 months	6 months	9 months	12 months	
Eligibility checklist	x						
Trial discussed and PIS given	x						
Informed consent		x					
UTI questionnaire		x	x	x	x	x	x
Adverse events			x	x	x	x	x
SF-36		x	x	x	x	x	x
Resource use questionnaire			x	x	x	x	
Patient cost questionnaire						x	
Treatment satisfaction questionnaire						x	
Contingent valuation questionnaire						x	
Catheter specimens of urine (CSU) to central laboratory		x	x	x	x	x	x
Perianal swab		x		x		x	
Creatinine (eGFR) and LFT		x				x	

#### Screening: clinical records and face-to-face

General demographics and eligibility will be checked. Trial information will be provided to the participant and consent taken for randomisation, informing GP of participation in trial, contact by researcher for semi-structured interview at end of trial participation (12 months), sending urine and perianal swab specimens to central laboratory 6 months after trial completion (18 months), access by research team to clinical records after active trial participation has ended and storage and use of blood, urine and perianal swab samples for further research.

If the patient is experiencing current symptomatic UTI, the infection will be treated first and the patient consented and randomised once symptom free. If the patient is already on antibiotic prophylaxis for UTI and agreeable to a 3-month washout period, the patient will be consented with a plan for randomisation in 3 months with frequent contact to ensure willingness to participate is maintained.

#### Baseline

The following procedures will take place at the baseline visit, which will be a face-to-face visit, after consent but prior to randomisation: baseline personal and health details, completion of SF-36 questionnaire, creatinine, LFT and creatinine clearance (via blood test or from clinical records if within 2 months of baseline) with storage of blood sample for future research, urine and perianal swab specimens for central microbiological analysis and storage, decision with regard to preferred means of questionnaire delivery, alerts and best time and means for contact by trial staff.

#### Randomisation

Randomisation will be performed as close as possible to the date of consent (normally immediately afterwards). Those participants willing to undergo a 3-month washout period will consent to the study at the beginning of the washout period, but will not be randomised or complete the other baseline measures until the washout period is complete, at which point their continued consent and eligibility for trial participation will be checked.

#### Post randomisation (discussion of trial documentation: face-to-face)

The participant log will be completed. Antibiotic prophylaxis will be discussed and prescribed (if allocated). The discussion will include what to do during a UTI, how this is defined and how the urine and perianal swab specimens are delivered to the central laboratory.

#### One month (after randomisation and repeated if required: telephone)

A member of the trial staff will contact the participant regarding general concerns, understanding of trial documentation and tolerance of prophylactic antibiotic agent (if allocated).

#### Three, six and nine months after randomisation (telephone/postal or face-to-face - according to individual participant circumstance)

The participant will complete trial outcome questionnaires and reports of any UTI and associated symptoms as well as adverse effects. Adherence to prophylaxis will be checked if allocated to this arm of the trial. During this time participants will complete the SF-36 and healthcare resource use questionnaire. They will send in a urine specimen during an asymptomatic period to the central laboratory for analysis and storage.

The local research staff will search clinical records (secondary and primary care) for episodes of UTI, episodes of UTI which are associated with fever greater than 38 °C, antibiotic prescription for UTI prophylaxis and other reasons, episodes of hospitalisation for UTI and other ill health and results of local laboratory urine culture.

#### During episode of symptomatic antibiotic-treated UTI (telephone/postal or face-to-face - according to individual participant circumstance)

The participant will complete a trial outcome questionnaire and report on the UTI, associated symptoms, adverse effects of treatment for UTI and continued adherence to prophylaxis (if allocated). In addition, participants will complete SF-36 questionnaires and send in a urine specimen to central and local laboratories prior to commencing a treatment course of antibiotics.

Local research staff will check clinical records for documented visits to healthcare for UTI, recorded fever greater than 38 °C, antibiotic prescription for UTI, hospitalisation and results of local laboratory urine culture.

#### Twelve months after randomisation (telephone or face-to-face - according to individual participant circumstance and local policy)

Participant will complete trial outcome questionnaires and report on the occurrence of UTIs and associated symptoms, adverse effects and adherence to prophylaxis (if allocated). In addition, participants will complete the SF-36 questionnaire, healthcare resource use questionnaire and patient costs (time and travel) questionnaire, as well as provide a urine (during asymptomatic period) and a perianal swab specimen which will be sent to the central laboratory. Creatinine and LFT (blood test) will be tested during asymptomatic periods. Finally the participants will complete a satisfaction with medication questionnaire and a contingent valuation questionnaire and will be asked to participate in a semi-structured interview (if selected and separately consented).

Local research staff will search clinical records (secondary and primary care) over 12 months of participation for episodes of UTI, episodes of UTI associated with fever greater than 38 °C, antibiotic prescriptions for UTI prophylaxis, UTI or other reasons, episodes of hospitalisation for UTI or other ill health as well as results of local laboratory urine culture.

Collection of outcome information by participant questionnaire may be onerous for some participants because of co-morbidities and disabilities linked to their underlying health condition. Local research staff, local clinical staff and, where appropriate, the carers of participants will be asked to help with completion of documentation for frequent contact with participants as is needed. The primary outcome will be validated if required from healthcare records. Full compliance with personal data protection will be ensured. We will balance provision of support for participants so that they adhere to their allocated intervention and record UTI events against avoidance of distress caused by the trial event schedule.

Urine and perianal swab specimens will be submitted regularly by participants during the trial to the central laboratory for selective culture of *E. coli*. These samples would not normally be collected during routine care, and the participants will be asymptomatic for UTI at the time of specimen collection. We therefore do not plan to inform the participant or the clinicians responsible for their care of the results. This policy would be sensitive to clinical concerns and exceptions readily made if, for example, the participant suffered an episode of severe sepsis and the results of the cultures would be helpful to clinical care. Similarly we will not routinely inform treating clinicians of the result of any positive culture from CSU sent to the central laboratory during an episode of symptomatic UTI. The reason for this is the delayed transit time and hence prolonged period before any result is available. This in most cases would make the result unhelpful to clinical care. Participants will be encouraged to also take a urine specimen to their treating clinician for local analysis.

#### Data handling and record keeping

Data will be recorded by site staff authorised by delegation log on electronic case report forms (eCRFs) in the clinical data management software package (MACRO™). Data transferred from site to the secure validated database by remote access will be secure and encrypted. Data will be handled, computerised and stored in accordance with the UK Data Protection Act 1998. Under the trial participant consent, identifiable data will be stored in a separate and limited access database to allow preparation and sending of follow-up documentation. The quality and retention of study data will be the responsibility of the Newcastle CTU. All study data will be retained in accordance with the latest directive on Good Clinical Practice (GCP) and local policy.

Clinical data will be entered into the database remotely at each site by the local investigator or another member of the site research team with delegated responsibility for this activity, together with data from questionnaires completed at face-to-face visits with participants. Questionnaires returned by post to the trial management office will be entered there. Trial management staff and database management staff will work closely with local site research teams to ensure that the data are as complete and accurate as possible. The Newcastle CTU will be responsible for checking and requesting missing data. Two reminders will be sent to participants for prompt return of the questionnaires. Extensive range and consistency checks will further enhance the quality of the data. Data collected during the course of the research will be kept strictly confidential and accessed only by members of the trial team. Patients will be allocated an individual specific trial number to allow anonymised versions of the secure database to be available to the trial team and subsequently more widely under open data access arrangements. The management system will be used to ensure trial correspondence is sent to each participant using their preferred mode of delivery. To comply with the fifth principle of the Data Protection Act 1998, personal data will not be kept for longer than is required for the purpose for which it has been acquired. The sponsor is responsible for ensuring that trial data are archived appropriately. Essential data will be retained for a period of at least 10 years following close of study in line with sponsor policy and the latest directive on GCP (2005/28/EC). Caldicott approval for use, transfer and storage of participant identifiable information will be obtained at each site.

#### Data sharing

Data will be archived in accordance with the Newcastle CTU standard operating procedure (SOP) and European Commission Directive 2005/28/EC Article 17 and made permanently available to the wider research community through deposition at the UK Data Archive. Research participants’ confidentiality will be protected through the removal of personal, confidential and sensitive data. In addition to data files (rendered as CSV-delimited text), data list files will provide descriptions of all variables, including how each variable was constructed and calculated where appropriate.

#### Discontinuation rules

The trial may be prematurely discontinued on the basis of new safety information or for other reasons given by the Data Monitoring Committee (DMC) and/or Trial Steering Committee (TSC), sponsor, regulatory authority or ethics committee concerned. The TSC will give advice on whether to continue or discontinue the study and make a recommendation to the funder and sponsor. If the study is prematurely discontinued, active participants will be informed and no further participant data will be collected.

### Study adherence and withdrawal

#### Assessment of adherence

Outcome data will be collected remotely whenever feasible by participant completion of postal or secure web-based questionnaires. This will be supplemented by e-mail or text alerts to participants notifying the need to complete questionnaires with additionally up to two reminders in these formats for non-responders. Local research staff will make use of planned routine clinical visits, whether for the underlying health condition or urinary tract monitoring, to check completion of trial documentation and collect clinical outcome information such as urine and perianal swab specimens. Participants allocated to prophylaxis will be contacted after 1 month to assess tolerability and if necessary allow change to alternative agent with re-checking of tolerability after a further month. We will assess adherence to the allocated arm (prophylaxis or no prophylaxis) by 3-monthly contact with the participant to check tolerability and surveillance of their primary healthcare record to record issuing of relevant prescriptions and consultations involving discussion of UTI treatment. We will contact participants more frequently if they need assistance to complete trial documents, particularly the UTI log.

All information will be recorded in the relevant CRF. If we do detect change from allocation, we will explore and record the reasons for this with the participant and the relevant clinician. Wherever possible these participants will remain on study and continue collection of planned outcome information. Trial literature will emphasise the need to adhere to the allocated strategy during the 12-month trial period if possible and will record any deviation. Multiple switching between prophylactic agents will be allowed. Previous studies suggest that this will affect approximately 12 % of participants [26], although a higher rate was seen in children (48 %) [27]. The trial statistician will monitor attrition rate against our anticipated maximum of 15 % and report to the TSC and DMC.

#### Withdrawal of participants

Participants have the right to withdraw from the study at any time for any reason, and without giving a reason. The investigator will also withdraw patients from the study intervention if it is judged to be in the patient’s best interests. It is understood by all concerned that an excessive rate of withdrawals can render the study uninterpretable; therefore, unnecessary withdrawal of patients should be avoided. Should a patient decide to withdraw from the study, all efforts will be made to report the reason for withdrawal as thoroughly as possible.

There are three withdrawal options:

1. Withdrawing completely (i.e. withdrawal from allocated treatment and consent to follow-up data collection)
2. Withdrawing from active participation in the trial but allowing continued review by the research team of healthcare records
3. Investigator-led participant change in status would be considered for the following reasons: Prophylaxis arm: Unable to tolerate any suitable antibiotic agent or pregnancy.
  No prophylaxis arm: Change in circumstance whereby starting prophylaxis is an urgent clinical necessity within 6 months of randomisation or pregnancy.

We will encourage participants who decide to withdraw to choose option 2, but if they wish to withdraw completely we will retain data collected up to the point of withdrawal. Participants will be asked if they would be happy for the reason for the decision to withdraw to be recorded. Participants who withdraw completely will not be replaced, but the rate of withdrawal will be monitored and reported to the DMC.

### Statistical analysis

An analysis of the primary outcome measure (incidence of UTI) for the full study will be performed both for the full data set and for the separate sub-groups defined by high and low baseline UTI rate (as specified during stratification for the randomisation process) using both the Poisson rate test and an incidence density ratio approach to allow for the different treatment durations; regression or generalised linear modelling approaches will be used to adjust for the effects of covariates. The model selection process will include the stratification factors (prior rate of UTI, presence of neurological disease and gender) and other baseline variables (person doing CISC [self or other], asymptomatic bacteriuria, age, type of catheter, use of antibiotic prophylaxis for UTI in the previous 12 months prior to randomisation and presence of bladder augmentation). The inclusion of interaction terms such as site will also be explored. The inclusion of baseline values as covariates will additionally enable the examination of possible interactions between effects observed and these values. Not all covariates mentioned above will be included in the final model, but all will be considered during the model selection process.

A number of additional secondary analyses will also be undertaken to examine the secondary outcome variables. Febrile and microbiologically confirmed UTI, asymptomatic bacteriuria and hospitalisation due to UTI will be analysed in a manner analogous to that for the primary outcome. Analyses of other outcomes will use similar regression or analysis of variance/covariance-based approaches as described above to compare between treatment groups while allowing for the effects of covariates. Baseline measures would be included in the analysis, either considered as possible covariates or by exploring changes in outcome measures from baseline. Analysis will be carried out primarily on an intention-to-treat basis, although other exploratory analyses such as per-protocol may also be considered. Data will be analysed at the end of the study; there are no planned interim analyses. Safety data will be regularly and frequently reviewed but will not be subjected to statistical analysis. Data with missing observations due to loss to follow-up will be examined to determine both the extent of the missing data and whether they are missing at random or are informative. If data are missing to a sufficient extent, the use of appropriate multiple imputation techniques will be considered.

### Economic analysis

Analyses will be carried out from the perspective of the UK NHS and personal and social services, but we will also take a wider perspective by including costs borne by the participants and their families. All unit costs will be derived using routine data sources [28] and study-specific estimates. Where appropriate, discounting will be applied to costs and outcomes at UK recommended rates [29]. Data on use of services will be combined with appropriate unit costs to produce a cost for each trial participant. From these a mean cost per intervention and a mean cost taking into account patient and carer costs will be estimated. Results for cost-effectiveness will be presented as point estimates of mean incremental costs and effects. The within-trial analysis will also compare changes in health-related quality of life (HRQoL) based on responses to the SF-36 and converted into the SF-6D [30] to estimate quality-adjusted life years (QALYs) using the area under the curve approach. They will subsequently be used in a cost-utility analysis based on avoidance of UTI. The results will be presented as point estimates of mean incremental costs and QALYs. Both cost-effectiveness and cost-utility analyses will include deterministic and stochastic sensitivity analyses, presented as point estimates and cost-effectiveness acceptability curves.

A contingent valuation study at 12 months will collect individuals’ willingness to pay for a reduction in the number of UTIs. For a given level of income, higher monetary values indicate that they would derive greater benefit. This method will enable us to place a monetary value on the health outcome, going beyond the QALY framework, and also to conduct a cost-benefit analysis. The cost-benefit analysis expresses both costs and benefits in commensurate units, which enables a comparison to be made between strategies [31]. The decision rule for the cost-benefit analysis is therefore relatively simple: if the benefits measured in pounds sterling (£) exceed the costs, this represents a gain in welfare and the strategy is deemed worthwhile [32]. Results will be presented as incremental net benefits (net benefits = mean willingness to pay – mean cost of intervention). Both stochastic and deterministic sensitivity analyses will be conducted and the results presented as incremental net benefit curves and as the probability that each treatment would be considered cost-effective.

### Sample size calculation

We plan to recruit 372 participants to the trial. Based on systematic reviews [11, 14] and expert (including patient) opinion, we believe that an overall 20 % reduction in symptomatic UTI rate from an average of 3 to 2.4 episodes per year represents the minimum clinically important difference. Using the Poisson rate test, completion of the study by 158 participants in each arm, 316 in total, would give 90 % power to detect this difference at the 5 % level. A total of 372 would allow for a 15 % attrition rate estimated from previous trials included in the systematic review. The attrition rate will be monitored and the sample size increased if necessary. Half this sample size would give 92 % power to detect a 25 % difference in the high frequency sub-group (from 4 to 3 episodes per year) and more than 99 % power for a 50 % reduction in the low frequency group (from 2 to 1) without allowance for multiple testing. We will approach approximately 750 eligible patients, anticipating a 50 % recruitment rate. We are currently recruiting from 52 sites which are coordinated through seven trial hubs. This number will be adjusted according to site and overall recruitment rate. For the qualitative sub-study of participant perception of benefit we will interview 30 trial participants who, at the time of trial consent, expressed a willingness to be interviewed at completion. We will create a purposive sample of approximately 20 participants from the prophylaxis arm and 10 from the no prophylaxis arm including some with neurological disease and some who did not complete the trial as allocated. We anticipate this will be sufficient to saturate themes arising from qualitative analysis of interview transcripts. For contingent valuation we will use a sample of 100 participants in each group; 200 in total will complete an exit questionnaire.

#### Qualitative analysis of participant exit interviews

We will use qualitative methodology to conduct and analyse semi-structured interviews with a purposive sample of trial participants to explore their views and experiences of self-catheterisation, the impact of UTI on their well-being, attitudes towards use of antibiotic prophylaxis and adherence to this treatment. This will inform interpretation of measures of effectiveness, particularly regarding adherence, and will also add insights that can further refine implementation of the intervention into practice. The sample will be weighted towards the prophylaxis arm in order to better explore the comparative experiences of this intervention for people with neurological and non-neurological underlying conditions. Interviews lasting approximately 45 minutes will be conducted by telephone with development of the schedule conducted in an iterative fashion in order to follow up unanticipated themes. Constant comparison techniques to check experiences against those of others in the sample will be used to ensure that the analysis represents all perspectives.

Interviews will be recorded, transcribed verbatim, anonymised and stored electronically with restricted access. Data will be transcribed and uploaded into filing software and coded for recurrent themes drawing on a framework analysis approach [33]. Transcripts will be charted, classified and organised according to key themes, concepts and emergent categories. The analytic matrix will include key attributes such as antibiotic use, sex, degree of adherence and neurological/non-neurological condition and will facilitate cross-referencing attributes with nodes/themes. Transcripts will be read by more than one researcher and discussed with the wider team to establish a rigorous analytical framework to find true patterns in the data. This will go beyond description to evaluate meanings of participants’ experiences in greater depth. Negative cases will be sought and further interrogated to explore the reasons for variation of experience or views and unanticipated themes will be searched for [34, 35]. A sample (*n* = 5) of coded transcripts will be checked and verified by a second researcher to ensure reliability.

### Study monitoring

Quality control will be maintained through adherence to SOPs published by the Newcastle CTU, study protocol, the principles of GCP, research governance and clinical trials for investigational medicinal products regulations. An independent Data Monitoring and Ethics Committee (DMC) has been convened to undertake independent review. The purpose of this committee will be to monitor efficacy and safety endpoints, and it will operate according to written terms of reference linked to the DAMOCLES charter [35]. Only the DMC will have access to full unblinded study data, if requested, prior to completion of the trial. All analyses will follow a carefully documented statistical analysis plan. The TSC and DMC will be asked to review and comment on this plan prior to analysis. A single main analysis will be performed at the end of the trial when all follow-up has been completed. The DMC will meet initially to agree on terms of reference and other procedures. The final trial report will contain full details of the analytical methodology. The DMC will meet at least three times: at the start, middle and completion of the study. At the first meeting, the committee will agree on its charter of operation and discuss and advise on criteria for the need for interim analysis and adoption of a formal set of stopping rules for efficacy or safety.

A Trial Steering Committee (TSC) has been established to provide overall supervision of the trial. The committee will meet approximately every 6 months during recruitment and annually thereafter for the duration of the trial.

Monitoring of study conduct and collected data will be performed by a combination of central review and focused site monitoring visits to ensure the study is conducted in accordance with GCP. Study site monitoring will be undertaken by the central trial management team. The main areas of focus will include validity of consent, serious adverse events and essential documents in study. Site monitoring will include review of all original consent forms as part of the study file. Confirmation of the presence of a copy in the patient hospital notes may be requested for 10 % of participants, and comparison of all original consent forms against the study participant identification list, verification of all reported serious adverse events against clinical records (source data verification), the presence of essential documents in the Investigator Site File and study files and verification of primary endpoint data and eligibility data for 10 % of participants entered in the study may be requested. Central monitoring will include review of applications for study authorisations and submissions of progress/safety reports for accuracy and completeness prior to submission, review of all documentation essential for study initiation prior to site authorisation and statistical monitoring for outlier sites and unusual data patterns.

All monitoring findings will be reported and followed up with the appropriate personnel in a timely manner. The study may be subject to inspection and audit by the Research and Development Directorate, Newcastle upon Tyne Hospitals NHS Foundation Trust, under their remit as sponsor, and by other regulatory bodies to ensure adherence to GCP. The site investigators and their institutions will permit trial-related monitoring, audits, REC review and regulatory inspection, providing direct access to source data/documents.

### Ethical approval and confidentiality

The conduct of this study will be in accordance with the recommendations for physicians involved in research on human subjects adopted by the 18th World Medical Assembly, Helsinki 1964 and later revisions. Favourable ethics opinion covering recruitment across all UK NHS sites was obtained from National Research Ethics Service Committee North East - Sunderland on 1 August 2014 (reference: 13/NE/0196). Local approvals were obtained from the Newcastle upon Tyne Hospitals NHS Foundation Trust, Northumberland Tyne and Wear NHS Foundation Trust, Newcastle Primary Care Trust, City Hospital Sunderland NHS Foundation Trust, Mid Yorkshire Hospitals NHS Trust, Leeds Community Healthcare NHS Trust, North Bristol NHS Trust, Bristol Primary Care Trust, Bath and North East Somerset Primary Care Trust, Weston Area Health NHS Trust, Cambridge University Hospitals NHS Foundation Trust, Southampton University Hospital NHS Trust, NHS Greater Glasgow and Clyde, SPCRN North, County Durham Primary Care Trust, Dorset Primary Care Trust, Hampshire Primary Care Trust, Solent NHS Trust, Leeds Teaching Hospitals NHS Trust, Leeds Community Healthcare NHS Trust, Ipswich Hospital NHS Trust, Southport and Ormskirk Hospital NHS Trust, University Hospital Birmingham NHS Foundation Trust, Gloucestershire Hospital NHS Foundation Trust, Gloucestershire Primary Care Trust, South Tees Hospitals NHS Foundation Trust, Royal Bolton Hospital NHS Foundation Trust, NHS Tayside, Royal National Orthopaedic Hospital NHS Trust, Guy's and St Thomas’ NHS Foundation Trust, NHS Lothian, University Hospitals Coventry and Warwickshire NHS Trust, NHS Ayrshire & Arran, Sheffield Teaching Hospitals NHS Foundation Trust, East Kent Hospitals University NHS Foundation Trust, The Royal Wolverhampton Hospitals NHS Trust, Imperial College Healthcare NHS Trust, Bedford Hospital NHS Trust, Salford Royal NHS Foundation Trust, NHS Highland, Northern Devon Healthcare NHS Trust and North Lancashire Teaching Primary Care Trust R&D committees.

#### Informed consent

All participants will undergo a process of informed consent which will include the delivery of balanced written information concerning the need and overall benefit of the trial followed up by discussion with a local trial coordinator. This discussion will include a check of understanding concerning benefits and risks of participation and ensuring that participants accept that the treatment will be allocated at random regardless of any personal preference they may have. Participants will be free to withdraw their consent at any time, and if this happens they will be given the opportunity to withdraw their data collected up to that time.

#### Compliance with Medicines for Human Use (Clinical Trials) Regulations

We have made a risk assessment of the potential hazards associated with this trial including those occurring and resulting in harm to the participants or researchers. The investigational medicinal products (IMPs) to be used in the trial are all licensed in dosage and form for use in prophylaxis against UTI in the UK and are standard care for this indication [23–25]. We thus judge that from an IMP perspective there is low risk to trial participants. Apart from the intervention in the experimental arm, participants in both arms of the trial will be subject to routine clinical care only, and we therefore consider that risks other than those related to the IMP are also low. Risks associated with the design and methods of the trial including the clinical procedures specified in the protocol, participants’ rights related to consent and protection of data and the reliability of trial results have also been assessed. The robust design of the study to mitigate and manage these risks has led to the decision, supported by the sponsor, to be granted ‘Type A’ status (low risk - notification only) from the UK Medicines and Healthcare Products Regulatory Agency (MHRA), allowing for a risk-proportionate trial management and monitoring approach to the trial. A structured safety monitoring plan will be made to assess risk management by all relevant parties including the sponsor, regulators, pharmacists and regulatory and governance staff. This was submitted to the MHRA along with the notification application.

#### Confidentiality

Personal data will be regarded as strictly confidential. The study will comply with the Data Protection Act, 1998. All study records and Investigator Site Files will be kept at site in a locked filing cabinet with restricted access. All trial laboratory samples will be labelled with a unique study identification number and patient date of birth only (linked in anonymised form).

#### SPIRIT

This protocol has been written in accordance with the Standard Protocol Items: Recommendations for Interventional Trials (SPIRIT) guidelines. Please refer to the SPIRIT checklist that was submitted alongside this publication for further details (see Additional file 1).

## Discussion

This study is a pragmatic patient-oriented trial aiming to capture a true representation of the actual patient population. For this reason it was decided to not discriminate against specific catheter types. In addition, inclusion/exclusion criteria were chosen to allow the capturing of the relevant patient group. The importance of this is also reflected in the mixed recruitment from both primary and secondary care sites, which will aid in achieving a balanced patient population.

This trial seeks to follow standard local patterns and pathways of care with the only additional intervention being randomisation between the two strategies under test and collection of baseline and outcome information. Given the degree of uncertainty regarding the use of antibiotic prophylaxis against UTI in this patient group, there is as yet no definitive evidence that randomisation to either arm will result in greater benefit or harm for participants; this being the reason for the trial.

The use of antibiotic prophylaxis may be associated with adverse effects related to individual agents or changes to normal bacterial flora. These risks will be minimised by carefully worded trial information given to participants randomised to prophylaxis and their clinicians to enable selection of the individually most appropriate agent and information concerning use of oral probiotics. The order of preference of prophylactic agents we will advise, together with guidance to avoid the use of other agents, will ensure that the great majority of participants will be able to use one of the three recommended agents and switch between them if necessary. We have planned what we believe is a sufficiently comprehensive but feasible program of bacterial surveillance that will detect potentially serious changes to bacterial ecology in the trial groups. The main benefit will be to resolve uncertainty concerning the effectiveness of antibiotic prophylaxis in this group, thereby reducing variation in practice.

### Funder statement

This article/paper/report presents independent research funded by the National Institute for Health Research (NIHR). The views expressed are those of the author(s) and not necessarily those of the NHS, the NIHR or the UK Government’s Department of Health.

## Trial status

The AnTIC study is currently recruiting in 52 UK research centres. The first patient was randomised on 26 November 2013, and recruitment is anticipated to end in January 2016 with follow-up completed in February 2017.

## Abbreviations

ACA, Association for Continence Advice; BAUS, British Association of Urological Surgeons; BIA, British Infection Association; BNF, British National Formulary; BSUG, British Society of Urogynaecology; CDC, Centers for Disease Control and Prevention; CI, confidence interval; CISC, clean intermittent self-catheterisation; CLRN, Comprehensive Local Research Network; CRC, Clinical Research Collaboration; CSU, catheter specimens of urine; CTiMP, Clinical Trial of an Investigational Medicinal Product; CTU, Clinical Trials Unit; DMC, Data Monitoring Committee; *E. coli*, *Escherichia coli*; eCRF, electronic case report form; eGFR, estimated glomerular filtration rate; EU, European Union; GCP, Good Clinical Practice; GP, general practitioner; HRQoL, health-related quality of life; HTA, Health Technology Assessment (Programme); IMP, investigational medicinal product; LFT, liver function test; MHRA, Medicines and Healthcare Products Regulatory Agency; NHS, National Health Service; NICE, National Institute for Health and Care Excellence; NIHR, National Institute for Health Research; NIHR CSP, National Institute for Health Research Coordinated System for gaining NHS Permission; PCRN, Primary Care Research Network; PI, Principal Investigator; PIC, Participant Identification Centre; QALY, quality-adjusted life year; QoL, quality of life; RCT, randomised controlled trial; R&D, Research and Development (departments of NHS Trusts); RUTI, recurrent urinary tract infection; SF-12, Medical Outcomes Short Form 12-item questionnaire; SF-36, Medical Outcomes Short Form 36-item questionnaire; SmPC, Summary of Product Characteristics; SOP, standard operating procedure; TMG, Trial Management Group; TSC, Trial Steering Committee; UK, United Kingdom; UTI, urinary tract infection; WTP, willingness to pay

## Additional file

Additional file 1: AnTIC protocol trials publication SPIRITchecklist. (DOC 119 kb)

## Footnotes

1 The definition chosen was the laboratory report of one or two isolates of a uropathogen at ≥10^5^ cfu/mL or a single isolate at ≥10^4^ cfu/mL in a symptomatic participant [22]. The central laboratory result will be used preferentially for this outcome with the local result used only if a suitable sample was not received by the central laboratory.
